# Sex differences in the predictive value of insulin resistance surrogate indicators for prediabetes among Chinese adults aged 18–45 years: a multicenter cohort study

**DOI:** 10.3389/fendo.2026.1872088

**Published:** 2026-06-24

**Authors:** Yafei Chang, Luming Zhang, Shafiu Adam UmarShinge, Rui Gu, Xingchao Zhou, Qingzhou Zhang, Xinyi Zhang, Binbin Zhang, Jiawei Liu, Yanqi Yang

**Affiliations:** 1 Department of Cardiovascular Surgery, The Eighth Affiliated Hospital of Sun Yat-sen University, Shenzhen, Guangdong, China; 2 Department of Intensive Care Unit, The First Affiliated Hospital of Jinan University, Guangzhou, China; 3 Zhongshan School of Medicine, Sun Yat-sen University, Guangzhou, China; 4 Department of Cardiothoracic and Vascular Surgery, University Hospital, Linköping University, Linköping, Sweden

**Keywords:** Chinese population, insulin resistance, METS-IR, sex differences, TyG index

## Abstract

**Objective:**

The aim of this study was to evaluate and compare the predictive value of four insulin resistance (IR) surrogate indices for incident prediabetes and examine sex-specific disparities in their predictive performance.

**Methods:**

This multicenter retrospective cohort study enrolled 63,795 adults aged 18–45 years (51.2% male) with normoglycemia at baseline from the Rich Healthcare Group Database (2010–2016). Participants were followed for a median of 2.97 years. Cox proportional hazards regression models, restricted cubic spline analyses, and receiver operating characteristic (ROC) curve analysis were employed to assess associations and discriminative performance. Statistical analyses were conducted using R software and EmpowerStats.

**Results:**

During follow-up, 5,304 (8.31%) participants developed prediabetes, with a significantly higher incidence in men than in women (10.76% vs. 5.74%; *p*< 0.001). After multivariable adjustment, all four IR indices were independently associated with incident prediabetes. Sex-stratified analyses demonstrated markedly stronger associations in women, particularly for metabolic score for insulin resistance (METS-IR) [fully adjusted hazard ratio (HR): 7.82, 95% confidence interval (CI): 5.81–10.51 vs. 1.45, 95% CI: 1.18–1.77 in men]. Triglyceride-glucose–body mass index (TyG-BMI) demonstrated the highest predictive accuracy in the overall population [area under the curve (AUC) = 0.6497], and women exhibited superior discriminative performance across all indices compared to men.

**Conclusion:**

IR surrogate indices demonstrate significantly greater predictive value for prediabetes in young Chinese women than in men, with METS-IR exhibiting the most pronounced sex disparity. These findings support sex-specific risk stratification using routine IR surrogate indices in prediabetes screening.

## 1 Introduction

The global burden of prediabetes has reached epidemic proportions, representing a critical window of opportunity for preventing progression to overt type 2 diabetes mellitus (T2DM) and associated cardiovascular complications (1). Defined by impaired fasting glucose (IFG) and/or impaired glucose tolerance (IGT), prediabetes is estimated to affect approximately 21% of the global adult population (2, 3). In China, the prevalence of prediabetes has risen dramatically in recent years, with recent national surveys indicating that 35.2% of adults are in the prediabetic stage, implying that over one-third of Chinese adults stand at the threshold of diabetes (4, 5). The trajectory among younger populations is of particular concern, underscoring the urgent need for early identification of high-risk young adults before irreversible metabolic deterioration occurs (5, 6).

Insulin resistance (IR) is the core pathophysiological driver of prediabetes, typically manifesting years before detectable glycemic abnormalities emerge, and independently predicts future cardiovascular events (7). The hyperinsulinemic–euglycemic clamp remains the gold standard for quantifying IR; however, its cost, technical complexity, and invasive nature limit its practical applicability in large-scale population screening or longitudinal surveillance (8). Simple, cost-effective surrogate markers derived from routine biochemical and anthropometric measurements have therefore emerged as practical alternatives. Among these, the triglyceride-glucose index (TyG), derived from fasting glucose and triglyceride concentrations, has demonstrated robust associations with IR and incident T2DM across diverse populations (9, 10). Composite indices including TyG-body mass index (TyG-BMI), atherogenic index of plasma (AIP), and metabolic score for insulin resistance (METS-IR) have shown incremental predictive value over the TyG index alone for metabolic disorders (11–13).

Evidence from epidemiological studies has documented a higher prevalence of IFG in men, attributable to hepatic IR and early impairment of insulin secretion (14). These biological differences extend to the predictive utility of metabolic markers. Research indicates that T2DM is more accurately predicted in women than in men using multiple anthropometric and biochemical measures, highlighting the importance of sex-based subgroup analysis in diabetes research (15). Recent studies demonstrate that the TyG index exhibits stronger predictive value for T2DM in women than in men, and that METS-IR exhibits sex-specific nonlinear associations with glucose metabolism (16, 17). Nevertheless, most prior investigations of IR surrogate indicators have focused on T2DM rather than prediabetes as the primary outcome, and few have systematically characterized the sex-specific predictive value of these indices in young Chinese populations.

This multicenter retrospective cohort study utilizing the Rich Healthcare Group Database was designed to evaluate the predictive performance of four IR surrogate indices (TyG index, TyG-BMI, AIP, and METS-IR) for incident prediabetes in young Chinese adults, with particular emphasis on quantifying sex differences in their prognostic utility. We hypothesize that these indices exhibit differential predictive capacity in male versus female patients, which may inform the development of sex-stratified screening protocols and targeted preventive strategies.

## 2 Methods

### 2.1 Data source and study population

This multicenter retrospective cohort study utilized data from the Rich Healthcare Group Database, a computer-based health examination system comprising medical records of participants undergoing health check-ups at multiple centers across China between 2010 and 2016 described in detail previously (18). The initial dataset included 685,277 participants aged ≥20 years with at least two health examination visits during the study period. Participants were sequentially excluded based on the following criteria: missing weight or height measurements (*n* = 103,946); missing sex data (*n* = 1); extreme body mass index (BMI) values (<15 kg/m² or >55 kg/m², *n* = 152); missing baseline fasting plasma glucose (FPG) measurements (*n* = 31,370); visit intervals less than 2 years (*n* = 324,233); diabetes mellitus at baseline, defined as self-reported diabetes, use of glucose-lowering medications, or FPG ≥ 7.0 mmol/L (*n* = 7,112); and indeterminate diabetes status at follow-up (*n* = 6,630). These sequential exclusions yielded an intermediate analytical cohort of 211,833 individuals.

**Figure 1:**
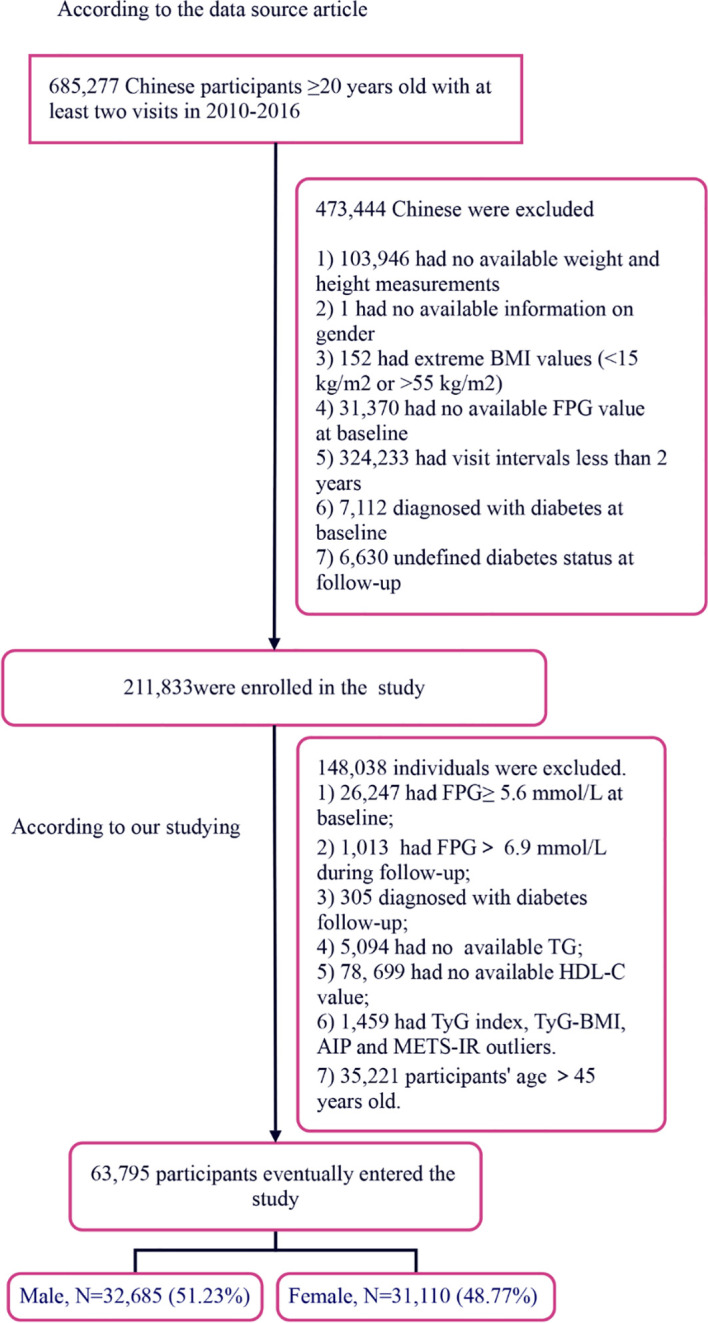
Flowchart of the study participants in the Chinese population.

For the present analysis, the population was further restricted to young adults aged ≤45 years with normoglycemia at baseline (6). After applying these criteria, a total of 63,795 participants were included in the final analysis. The study flowchart is presented in **Figure 1**.

This study was conducted in accordance with the Declaration of Helsinki. The Rich Healthcare Group Database is de-identified and publicly available for research use; accordingly, the requirement for individual informed consent was waived. The study protocol was approved by the Institutional Review Board (IRB).

### 2.2 Data collection and measurements

All participants underwent standardized health examinations performed by trained medical personnel. Anthropometric measurements included height, weight, and blood pressure. BMI was calculated as body weight (kg) divided by the square of height (m²). Systolic blood pressure (SBP) and diastolic blood pressure (DBP) were measured using calibrated sphygmomanometers following standardized protocols (18).

Fasting venous blood samples were collected after a minimum 8-h overnight fast. Biochemical assays, including FPG, total cholesterol (TC), triglycerides (TG), high-density lipoprotein cholesterol (HDL-C), low-density lipoprotein cholesterol (LDL-C), alanine aminotransferase (ALT), blood urea nitrogen (BUN), and serum creatinine (Scr), were performed using standard automated analyzers in accordance with manufacturer protocols (18).

### 2.3 Definition of insulin resistance surrogate indices

The four IR surrogate indices were calculated using the following formulas:


TyG index = ln [TG (mg/dL) × FPG (mg/dL)/2]

(19)


TyG−BMI = TyG index × BMI

(20)


AIP (atherogenic index of plasma) = Log [TG (mmol/L)/HDL-C (mmol/L)]

(21)


METS−IR (metabolic score for insulin resistance) = Ln [(2 × FPG (mg/dL) + TG (mg/dL)) × BMI/Ln(HDL−C (mg/dL))]

(13)

### 2.4 Outcome ascertainment

The primary outcome was incident prediabetes during follow-up. Prediabetes was defined in accordance with the American Diabetes Association criteria (22): FPG 5.6–6.9 mmol/L (100–125 mg/dL) at the follow-up visit.

### 2.5 Statistical analysis

Baseline characteristics were summarized stratified by sex and prediabetes status. Normally distributed continuous variables are expressed as mean ± standard deviation (SD) and compared using Student’s *t*-test. Skewed continuous variables are expressed as median [interquartile range (IQR)] and compared using the Mann–Whitney *U* test. Categorical variables are expressed as frequencies and proportions and compared using the chi-square test.

Cox proportional hazards regression was used to assess the associations between IR surrogate index (as continuous variables) and incident prediabetes. To evaluate multicollinearity, variance inflation factors (VIFs) were calculated for all covariates; all VIF values were<5, indicating the absence of significant collinearity (**Supplementary Table 1**). Taking follow-up duration as the time scale, we established multivariable Cox proportional hazards regression models with hierarchical covariate adjustment. Three sequentially adjusted models were constructed to explore the relationship between IR surrogate indicators and new-onset prediabetes, as well as to quantify the confounding effects of relevant covariates.

Model I (unadjusted): IR index as the sole independent variable.

Model II (partially adjusted): Adjusted for age.

Model III (fully adjusted): Adjusted for age, SBP, DBP, ALT, BUN, Scr, smoking status, drinking status, and family history of diabetes.

Hazard ratios (HRs) and 95% confidence intervals (CIs) are reported per one-unit increment for each index with the exception of TyG-BMI, which is reported per 10-unit increment. To evaluate potential nonlinear associations between each IR index and incident prediabetes, restricted cubic spline analysis was performed with three knots in the fully adjusted model. Likelihood ratio tests were used to compare models with and without nonlinear terms. Where significant nonlinearity was detected, two-segment linear regression was applied to identify the inflection point (*K*), with HRs estimated separately below and above the threshold.

Receiver operating characteristic (ROC) curve analysis was used to evaluate the discriminative ability of each IR index for predicting incident prediabetes, with area under the curve (AUC) values calculated. To rank candidate predictors, the Boruta algorithm, a random forest-based feature selection method that creates shadow variables and identifies those with *Z*-scores significantly exceeding random noise, was applied.

Three pre-specified sensitivity analyses were conducted to assess the robustness of the findings. First, exclusion of participants with follow-up duration< 3 years to address potential reverse causality. Second, exclusion of participants aged > 60 years, to confirm the age restriction applied in the primary analysis. Third, exclusion of participants with baseline SBP ≥ 140 mmHg, to asses potential confounding by hypertension.

All statistical analyses were performed using R (R Foundation for Statistical Computing, Vienna, Austria) and EmpowerStats (X&Y Solutions, Boston, MA). All tests were two-sided, and *p*-value< 0.05 was considered statistically significant.

## 3 Results

### 3.1 Baseline characteristics

This study included 63,795 young adults (aged 18–45 years) with normoglycemia at baseline, comprising 51.2% men and 48.8% women, with a median follow-up of 2.97 years. During follow-up, 8.31% progressed to prediabetes, with a significantly higher incidence in men than in women (10.76% vs. 5.74%, *p*< 0.001) (**Table 1**).

**Table 1:** Participant characteristics stratified by gender.

Characteristics	Total (*n* = 63,795)	Male (*n* = 32,685)	Female (*n* = 31,110)	*p*-value
Age, years	35.12 ± 5.28	35.06 ± 5.21	35.18 ± 5.35	0.004
BMI, kg/m^2^	22.60 ± 3.17	23.81 ± 3.10	21.34 ± 2.73	<0.001
SBP, mmHg	114.65 ± 13.83	119.67 ± 13.33	109.36 ± 12.30	<0.001
DBP, mmHg	71.75 ± 9.88	74.55 ± 9.79	68.80 ± 9.08	<0.001
FPG, mmol/L	4.75 ± 0.48	4.77 ± 0.49	4.72 ± 0.46	<0.001
TC, mmol/L	4.58 ± 0.82	4.66 ± 0.84	4.50 ± 0.78	<0.001
TG, mmol/L	1.16 ± 0.72	1.42 ± 0.82	0.89 ± 0.47	<0.001
HDL-C, mmol/L	1.39 ± 0.30	1.29 ± 0.26	1.48 ± 0.30	<0.001
LDL-C, mmol/L	2.64 ± 0.63	2.71 ± 0.65	2.56 ± 0.60	<0.001
ALT, U/L	16.90 (12.00–26.40)	23.40 (16.60–35.10)	12.80 (10.00–16.80)	<0.001
BUN, mmol/L	4.48 ± 1.09	4.78 ± 1.08	4.16 ± 1.01	<0.001
Scr, μmol/L	69.28 ± 15.40	80.64 ± 11.04	57.34 ± 8.93	<0.001
TyG index	8.23 ± 0.55	8.45 ± 0.54	8.00 ± 0.46	<0.001
TyG-BMI (×10)	18.68 ± 3.41	20.17 ± 3.34	17.12 ± 2.72	<0.001
AIP	−0.16 (−0.33 to 0.05)	−0.02 (−0.20 to 0.16)	−0.28 (−0.42 to −0.12)	<0.001
METS-IR	2.20 ± 0.17	2.27 ± 0.16	2.13 ± 0.14	<0.001
Smoking status, *n* (%)				<0.001
Current smoker	7,826 (12.27%)	7,783 (23.81%)	43 (0.14%)	
Ever smoker	2,104 (3.30%)	2,062 (6.31%)	42 (0.14%)	
Never smoker	53,865 (84.43%)	22,840 (69.88%)	31,025 (99.73%)	
Drinking status, *n* (%)				<0.001
Current drinker	855 (1.34%)	802 (2.45%)	53 (0.17%)	
Ever drinker	9,372 (14.69%)	8,438 (25.82%)	934 (3.00%)	
Never drinker	53,568 (83.97%)	23,445 (71.73%)	30,123 (96.83%)	
Family history of diabetes, *n* (%)				<0.001
No	62,289 (97.64%)	32,129 (98.30%)	30,160 (96.95%)	
Yes	1,506 (2.36%)	556 (1.70%)	950 (3.05%)	
Prediabetes accident				<0.001
No	58,491 (91.69%)	29,167 (89.24%)	29,324 (94.26%)	
Yes	5,304 (8.31%)	3,518 (10.76%)	1,786 (5.74%)	
Follow-up (years)	2.97 (2.12–3.95)	2.97 (2.12–3.93)	2.98 (2.12–3.95)	0.005

Values are *n* (%), means or medians (quartiles).

BMI, body mass index; FPG, fasting plasma glucose; SBP, systolic blood pressure; DBP, diastolic blood pressure; TC, total cholesterol; TG, triglycerides; HDL-C, high-density lipoprotein cholesterol; LDL-C, low-density lipoprotein cholesterol; ALT, alanine aminotransferase; BUN, blood urea nitrogen; Scr, serum creatinine; TyG, triglyceride-glucose index; TyG-BMI, triglyceride-glucose–body mass index; AIP, atherogenic index of plasma; METS-IR, metabolic score for insulin resistance.

At baseline, men exhibited a substantially heavier metabolic burden: BMI, blood pressure, hepatic enzyme activity, and all four IR indices (TyG index, TyG-BMI, AIP, and METS-IR) were significantly higher in men than in women (all *p*< 0.001), and rate of smoking and alcohol consumption were markedly greater. When stratified by quartiles of each IR index, prediabetes incidence followed a clear dose–response gradient, rising significantly across ascending quartiles of the IR indices (e.g., TyG index 4.67% in Q1 vs. 13.61% in Q4, *p* for trend< 0.001), with a progressive decrease in the proportion of women across higher IR quartiles (**Table 1**).

When stratified by TyG index quartiles (**Supplementary Table 2**), participants with higher TyG levels were older and had higher BMI, blood pressure, glucose, lipids, and liver enzymes, with a progressively lower proportion of women (from 73.3% in Q1 to 21.0% in Q4), and the incidence of prediabetes increased from 4.67% to 13.61% across quartiles (*p* for trend< 0.001). Similar trends were observed across quartiles of TyG-BMI, AIP, and METS-IR (**Supplementary Tables 3**-**S5**).

As shown in **Supplementary Figure 1**, the TyG index ranged from 6.60 to 10.09 (mean, 8.23); TyG-BMI ranged from 107.70 to 299.40 (mean, 186.83); AIP ranged from −0.95 to 0.88, (median, −0.16); and METS-IR ranged from 1.71 to 2.75 (mean, 2.20).

### 3.2 Univariate analysis and risk factor importance ranking

Univariate Cox regression analysis (**Supplementary Table 6**) confirmed that all four IR indices were significantly associated with incident prediabetes (all *p*< 0.0001). METS-IR demonstrated the strongest unadjusted association (HR = 6.12, 95% CI: 5.29–7.08), followed by AIP (HR = 3.22, 95% CI: 2.94–3.53) and the TyG index (HR = 2.33, 95% CI: 2.22–2.43), consistent with prior studies identifying METS-IR and AIP as robust predictors of glucose metabolism disorders.

The Boruta algorithm was applied, to rank the importance of candidate predictors of prediabetes progression (**Supplementary Figure 2**). All four IR surrogate indices were confirmed as statistically important predictors with *Z*-scores substantially exceeding those of the corresponding shadow variables TyG-BMI and TyG index ranked relatively highest, consistent with the univariate regression. This machine learning-based validation corroborate the clinical relevance of IR surrogate indices as predictors of prediabetes risk.

### 3.3 Survival analysis

Kaplan–Meier survival curves (**Supplementary Figure 3**) demonstrated a clear dose–response relationship between the four IR indices and prediabetes progression. Participants in the highest quartile (Q4) of each index exhibited significantly higher cumulative incidence of prediabetes relative to those in the lowest quartile (Q1), with consistent gradients across all indices, further corroborating the positive association between IR severity and prediabetes risk.

### 3.4 Predictive value of IR indices for prediabetes risk and sex differences

Sex-stratified analyses revealed significant heterogeneity between men and women in the association between IR indices and incident prediabetes (**Table 2**). In the fully adjusted model, all four indices consistently demonstrated stronger predictive value in women than in men. METS-IR exhibited the most pronounced sex disparity: the HR in women was 7.82 (95% CI: 5.81–10.51), more than fivefold higher than that in men (HR = 1.45, 95% CI: 1.18–1.77; *p* for interaction< 0.001). Similarly, AIP demonstrated nearly twice the association strength in women compared with men (HR = 2.74, 95% CI: 2.27–3.31 vs. HR = 1.47, 95% CI: 1.29–1.68). TyG index and TyG-BMI also demonstrated stronger associations in women, though with smaller magnitudes of sex disparity.

**Table 2:** Associations between insulin resistance indices and the incident prediabetes in different models.

Group	Exposure	Model I (HR, 95% CI) *p*	Model II (HR, 95% CI) *p*	Model III (HR, 95% CI) *p*
	TyG index	2.33 (2.22, 2.43)<0.0001	2.29 (2.18, 2.40)<0.0001	1.90 (1.80, 2.00)<0.0001
All	TyG-BMI (×10)	1.15 (1.14, 1.16)<0.0001	1.15 (1.14, 1.16)<0.0001	1.11 (1.10, 1.12)<0.0001
(*N* = 63,795)	AIP	3.22 (2.94, 3.53)<0.0001	3.08 (2.81, 3.38)<0.0001	1.87 (1.68, 2.08)<0.0001
	METS-IR	6.12 (5.29, 7.08)<0.0001	5.76 (4.97, 6.67)<0.0001	2.62 (2.23, 3.09)<0.0001
	TyG index	1.95 (1.84, 2.07)<0.0001	1.93 (1.82, 2.05)<0.0001	1.78 (1.67, 1.89)<0.0001
Male	TyG-BMI (×10)	1.11 (1.10, 1.12)<0.0001	1.11 (1.10, 1.12)<0.0001	1.09 (1.08, 1.10)<0.0001
(*N* = 32,685)	AIP	1.92 (1.69, 2.16)<0.0001	1.83 (1.61, 2.07)<0.0001	1.47 (1.29, 1.68)<0.0001
	METS-IR	2.17 (1.78, 2.63)<0.0001	2.03 (1.67, 2.47)<0.0001	1.45 (1.18, 1.77) 0.0004
	TyG index	2.50 (2.29, 2.74)<0.0001	2.40 (2.19, 2.63)<0.0001	2.14 (1.95, 2.35)<0.0001
Female	TyG-BMI (×10)	1.19 (1.17, 1.21)<0.0001	1.18 (1.17, 1.20)<0.0001	1.16 (1.14, 1.18)<0.0001
(*N* = 31,110)	AIP	3.72 (3.10, 4.48)<0.0001	3.45 (2.87, 4.15)<0.0001	2.74 (2.27, 3.31)<0.0001
	METS-IR	12.45 (9.33, 16.60)<0.0001	11.15 (8.35, 14.90)<0.0001	7.82 (5.81, 10.51)<0.0001

Model I: we did not adjust other covariates.

Model II: we adjusted age.

Model III: we adjusted variables in Adjust II+ SBP, DBP, ALT, BUN, Scr, smoking status, drinking status, and family history of diabetes. HR, hazard ratio; CI, confidence interval.

Further notable observation was sex-specific attenuation of effect estimates across sequential adjustment models. In men, the HR for METS-IR decreased substantially from 2.17 (Model I) to 1.45 (Model III), suggesting a considerable proportion of the crude association in men is attributable to or mediated by other metabolic and lifestyle covariates. In women, although the HR for METS-IR similarly attenuated from 12.45 to 7.82, it remained markedly elevated, indicating robust independent predictive value not accounted for by the included covariates.

### 3.5 Nonlinear associations and inflection point analysis

**Figure 2:**
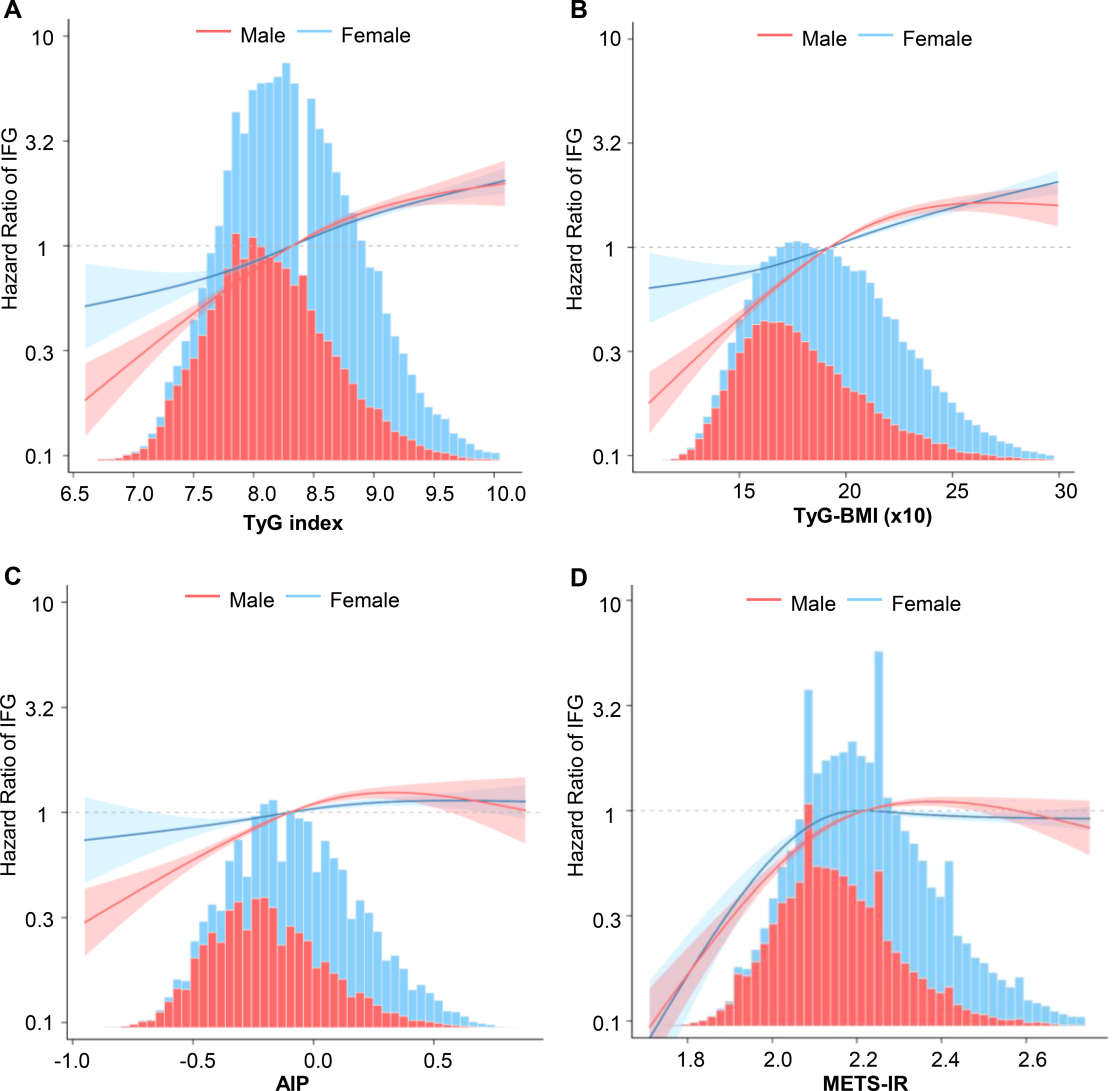
Non-linear relationships between insulin resistance indices and prediabetes risk.

Restricted cubic spline analysis revealed significant sex-specific differences in the nonlinear dose–response relationship between IR indices and incident prediabetes risk (**Figure 2**; **Table 3**). Among men, significant nonlinearity only for METS-IR (inflection point 2.14, *p*< 0.001), with risk increasing sharply below the threshold (HR = 104.18) and plateauing above it (HR = 0.88); the remaining three indices exhibited linear dose–response patterns. Women demonstrated a distinct pattern: TyG-BMI (inflection point 21.64), AIP (inflection point 0.14), and METS-IR (inflection point 2.26) all exhibit significant nonlinear associations (all *p*< 0.05), whereas the TyG index remained linear. These findings indicate that women manifest earlier and more multidimensional metabolic risk thresholds relative to men.

**Table 3:** Nonlinear associations between insulin resistance indices and prediabetes risk.

Gender	IR indices	TyG index	TyG-BMI (×10)	AIP	METS-IR
Male	Model I	1.78 (1.67, 1.89)<0.0001	1.09 (1.08, 1.10)<0.0001	1.47 (1.29, 1.68)<0.0001	1.45 (1.18, 1.77) 0.0004
	Model II				
	Inflection points	7.59	16.64	0.43	2.14
	<*K*	2.78 (1.06, 7.32) 0.0383	1.04 (0.96, 1.11) 0.3579	1.55 (1.33, 1.79)<0.0001	104.18 (28.96, 374.74)<0.0001
	≥*K*	1.76 (1.64, 1.88)<0.0001	1.09 (1.08, 1.10)<0.0001	0.76 (0.28, 2.05) 0.5937	0.88 (0.69, 1.13) 0.3199
	*p* for log-likelihood ratio test	0.350	0.199	0.180	<0.001
Female	Model I	2.14 (1.95, 2.35)<0.0001	1.16 (1.14, 1.18)<0.0001	2.74 (2.27, 3.31)<0.0001	7.82 (5.81, 10.51)<0.0001
	Model II				
	Inflection points	8.81	21.64	0.14	2.26
	<*K*	2.28 (2.03, 2.57)<0.0001	1.21 (1.18, 1.24)<0.0001	3.32 (2.62, 4.21)<0.0001	35.89 (21.36, 60.30)<0.0001
	≥*K*	1.53 (1.04, 2.27) 0.0316	1.05 (1.00, 1.10) 0.0336	0.88 (0.37, 2.13) 0.7844	0.76 (0.36, 1.60) 0.4702
	*p* for log-likelihood ratio test	0.074	<0.001	0.006	<0.001

Data were presented as HR (95% CI) *p-*value; Model I, linear analysis; Model II, non-linear analysis. Adjusted for age, SBP, DBP, ALT, BUN, Scr, smoking status, drinking status, and family history of diabetes. *K* indicates inflection point. *p* for log-likelihood ratio test< 0.05 indicates that Model II is significantly different from Model I.

Restricted cubic spline plots illustrate the dose–response association of each insulin resistance index with incident prediabetes. The solid red line represents the estimated hazard ratio (HR), and the red dashed shading indicates the 95% confidence interval. (A) TyG index; (B) TyG-BMI; (C) AIP; (D) METS-IR. *p*-values for non-linearity were<0.001 for all indices.

### 3.6 Predictive performance evaluation

ROC analysis demonstrated that TyG-BMI achieved the highest predictive accuracy in the overall population (AUC = 0.6497). Sex-stratified analyses revealed superior discriminative performance in women across all indices: TyG-BMI AUC = 0.6525 and METS-IR AUC = 0.6321 (sensitivity 69.2%) in women, compared with TyG-BMI AUC = 0.6019 and METS-IR AUC = 0.5636 in men. These findings are consistent with the Cox regression results and further validate the superior discriminative capacity of IR indices for prediabetes identification in women (**Supplementary Table 7**; **Supplementary Figure 4**).

### 3.7 Sensitivity analyses

Sensitivity analyses confirmed the robustness of the main findings (**Table 4**). After excluding participants with follow-up duration< 3 years (Model I), those aged > 60 years (Model II), and those with baseline SBP ≥ 140 mmHg (Model III), the direction and magnitude of associations between IR indices and prediabetes risk remained consistent with the primary analysis. Notably, in women, METS-IR consistently exhibited the strongest predictive performance across all sensitivity analyses (fully adjusted HR range: 7.82–8.34), while the effect size of METS-IR in men attenuated after excluding hypertensive participants, further supporting sex-specific pathophysiological differences.

**Table 4:** Associations between insulin resistance indices and the incident prediabetes in sensitive analysis.

Group	Exposure	Model I (HR, 95% CI) *p*	Model II (HR, 95% CI) *p*	Model III (HR, 95% CI) *p*
All (*N* = 99,016)	TyG index	2.03 (1.89, 2.18)<0.0001	1.90 (1.80, 2.00)<0.0001	1.91 (1.81, 2.02)<0.0001
	TyG-BMI (×10)	1.12 (1.11, 1.14)<0.0001	1.11 (1.10, 1.12)<0.0001	1.12 (1.11, 1.13)<0.0001
	AIP	2.35 (2.04, 2.72)<0.0001	1.87 (1.68, 2.08)<0.0001	1.91 (1.71, 2.12)<0.0001
	METS-IR	3.63 (2.90, 4.53)<0.0001	2.62 (2.23, 3.09)<0.0001	2.78 (2.34, 3.29)<0.0001
Male (*N* = 51,028)	TyG index	1.88 (1.72, 2.05)<0.0001	1.78 (1.67, 1.89)<0.0001	1.78 (1.66, 1.90)<0.0001
	TyG-BMI (×10)	1.10 (1.08, 1.11)<0.0001	1.09 (1.08, 1.10)<0.0001	1.09 (1.08, 1.10)<0.0001
	AIP	1.77 (1.48, 2.12)<0.0001	1.47 (1.29, 1.68)<0.0001	1.47 (1.29, 1.69)<0.0001
	METS-IR	1.88 (1.42, 2.48)<0.0001	1.45 (1.18, 1.77) 0.0004	1.48 (1.19, 1.84) 0.0003
Female (*N* = 47,988)	TyG index	2.27 (2.00, 2.59)<0.0001	2.14 (1.95, 2.35)<0.0001	2.17 (1.97, 2.39)<0.0001
	TyG-BMI (×10)	1.16 (1.14, 1.19)<0.0001	1.16 (1.14, 1.18)<0.0001	1.17 (1.15, 1.18)<0.0001
	AIP	3.51 (2.72, 4.53)<0.0001	2.74 (2.27, 3.31)<0.0001	2.82 (2.32, 3.42)<0.0001
	METS-IR	10.87 (7.29, 16.21)<0.0001	7.82 (5.81, 10.51)<0.0001	8.34 (6.15, 11.31)<0.0001

Model I: participants with<3 years of follow-up were excluded; covariates included age, SBP, DBP, ALT, BUN, Scr, smoking status, alcohol consumption, and family history of diabetes. Model II: participants aged >60 years were excluded; the same covariates as in Model I were adjusted. Model III: participants with baseline SBP ≥140 mmHg were excluded; identical covariates were adjusted. HR, hazard ratio; CI, confidence interval.

## 4 Discussion

In this large multicenter cohort study of 63,795 young Chinese adults with normoglycemia at baseline, we comprehensively evaluated the predictive performance of four IR surrogate indices—TyG index, TyG-BMI, AIP, and METS-IR—for incident prediabetes, with particular emphasis on sex-specific differences. Our study yielded four principal findings. First, all four IR indices were independently associated with incident prediabetes, with METS-IR demonstrating the strongest overall association. Second, the predictive value of all indices was substantially greater in women than in men, with METS-IR demonstrating the most pronounced sex disparity. Third, significant nonlinear dose–response relationships with sex-specific inflection points were identified for multiple indices. Fourth, TyG-BMI demonstrated the highest overall predictive accuracy, while women exhibited superior discriminative performance across all indices relative to men.

The positive associations between IR surrogate indices and incident prediabetes are consistent with prior investigations using prediabetes as the outcome. Feng et al., in a Chinese multicenter health examination cohort, reported that both TyG index and TyG-BMI were significantly associated with prediabetes risk (23). Xiao et al., in a 5-year retrospective cohort study of young Chinese adults, identified a nonlinear relationship between TyG index and prediabetes risk, with an inflection point at 9.39 (6). Jiang et al., using data from the China Health and Retirement Longitudinal Study (CHARLS), demonstrated a nonlinear positive association between AIP and both prediabetes and T2DM (24). The convergent findings across these investigations, spanning results from diverse study designs and populations, corroborate our findings and collectively support the utility of IR surrogate indices as practical tools for prediabetes risk screening.

The superior performance of METS-IR observed in our study is consistent with recent findings. Bello-Chavolla et al., who developed and validated METS-IR in a Mexican population, demonstrated that METS-IR complements and enhances traditional IR surrogates in predicting visceral adiposity and incident T2DM (13). A recent large-scale Chinese cohort study of 100,309 participants subsequently reported a positive association between METS-IR and prediabetes risk, with the critical observation that this association was stronger in women than in men (25). These findings collectively reinforce our observation that METS-IR is a robust, sex-sensitive predictor of dysglycemic risk.

The sex differences identified in our study represent a novel and clinically important contribution to the literature. Guo et al. reported that the TyG index had significantly greater predictive value for T2DM in women than in men, suggesting greater metabolic vulnerability to IR-mediated dysglycemia in women (26), warranting a sex-sensitive approach in diabetes risk assessment and preventive strategies (27). Our findings extend this sex disparity to encompass METS-IR and AIP, with METS-IR exhibiting a more than fivefold higher HR in women than in men. This pattern suggests that IR surrogate indices may capture distinct pathophysiological process across sexes and that sex-uniform threshold may substantially underestimate prediabetes risk in women.

Restricted cubic spline analyses revealed significant nonlinear dose–response relationships between several IR indices and incident prediabetes, with distinct sex-specific inflection points that carry important mechanistic and clinical implication. These findings add important nuance to the understanding of dose–response dynamics between IR and prediabetes. For AIP, an inflection point of 0.14 was identified in women; below this threshold, the association with prediabetes was particularly strong, whereas above it, the effect became non-significant, consistent with a saturation phenomenon. This saturation was absent in men, in whom the association between AIP and prediabetes did not exhibit significant threshold characteristics. Jiang et al., based on the CHARLS database, reported a nonlinear association between AIP and prediabetes, with risk increasing most steeply at lower AIP values; however, sex-stratified analysis of this specific nonlinear pattern has not been previously reported (24). The clinical implication is that, in women, interventions targeting AIP reduction may yield the greatest marginal risk reduction among individuals with values below the inflection point, whereas a linear risk model may be more appropriate for men. For METS-IR, significant threshold effects were observed in both sexes, with markedly elevated HRs below the inflection points suggesting that most of the excess prediabetes risk association with METS-IR is concentrated at lower values of this index. The substantially greater HR magnitude below the threshold in men than in women further underscores the sex-specific nature of METS-IR’s nonlinear predictive behavior. These threshold effects have important implications for clinical decision-making: individuals with METS-IR values just above the inflection point may be at disproportionately higher risk than those with values far above the threshold. For TyG-BMI, significant threshold effects were observed exclusively in women, consistent with prior reports of sex-specific predictive patterns for this index (28). The lack of a threshold effect in men may reflect a more uniformly linear BMI–metabolic risk relationship in this sex, or may be attributable to the substantially higher baseline BMI in men, which could obscure a discrete risk inflection point.

Several biological mechanisms may account for observed sex differences in the predictive utility of IR indices. Sex hormone regulation is a primary determinant: premenopausal women are typically more insulin-sensitive than age-matched men; consequently, elevation of IR surrogate indices in this population may signal disproportionately severe metabolic disruption. Estrogen exerts pleiotropic anti-diabetogenic effects through modulation of pancreatic beta-cell function, insulin signaling, and adipose tissue distribution, whereas androgens promote IR by suppressing insulin signaling and enhancing visceral adipogenesis. Differences in body fat distribution constitutes an additional mechanistic axis: men disproportionately accumulate visceral adiposity, while women tend to accumulate subcutaneous fat, which is metabolically less deleterious; however, when women develop visceral adiposity as reflected by elevated TyG-BMI and METS-IR, the downstream metabolic consequences may be more severe than in men with comparable absolute index value (29).

These findings support the adoption of sex-stratified screening models incorporating IR indices, particularly TyG-BMI and METS-IR. TyG-BMI, which integrates metabolic and anthropometric parameters derivable from routine health examinations without additional testing, represents a pragmatic first-line screening tool. The sex-specific inflection points identified through spline analyses provide quantitative, clinically actionable risk thresholds. Targeted lifestyle interventions should be prioritized for women with high METS-IR where the greatest risk reduction potential per unit change in the index is anticipated.

Several limitations require acknowledgement. First, the lack of direct insulin sensitivity measures (e.g., hyperinsulinemic–euglycemic clamp or HOMA-IR) prevented the validation of the IR surrogate indices against a gold standard. However, both TyG index and METS-IR have been previously validated against clamp measurements in independent populations. Second, prediabetes diagnosis was based solely on FPG rather than oral glucose tolerance testing, which may have led to systemic underdiagnosis of isolated IGT; FPG, nonetheless, is the most commonly employed criterion in large epidemiological studies and has high diagnostic specificity. Third, the absence of data on dietary intake, physical activity, and medication use, particularly oral contraceptives and hormone replacement therapy, represents a meaningful limitation in interpreting the magnitude of the observed sex differences. Fourth, prediabetes classification was mainly dependent on fasting blood glucose (FBG), since glycated hemoglobin (HbA1c) data were not consistently available. Given that HbA1c reflects long-term glycemic status and can identify more individuals with abnormal glycemic metabolism, future studies are warranted to incorporate HbA1c and oral glucose tolerance tests to achieve more comprehensive evaluation. In addition, the observed incidence rate of prediabetes (8.31%) should be interpreted in light of the study design, as it represents new-onset cases during follow-up rather than the overall population prevalence. Furthermore, the relatively young age of the study participants (aged 18–45 years) and the exclusion of individuals with prediabetes at baseline may partially account for the relatively low observed incidence.

## 5 Conclusions

In this large multicenter cohort of young Chinese adults with normoglycemia at baseline, TyG index, TyG-BMI, AIP, and METS-IR were each independently associated with incident prediabetes, with significant sex differences in their predictive performance. All four indices exhibited substantially stronger associations and superior discriminative capacity in women than in men, with METS-IR demonstrating the most pronounced sex disparity. TyG-BMI demonstrated the highest overall predictive accuracy. Collectively, these findings suggest that IR surrogate indices, particularly TyG-BMI and METS-IR, are valuable tools for prediabetes risk stratification in young adults and that sex should be explicitly incorporated when applying these markers in clinical screening practice. Prospective studies are warranted to validate the sex-specific risk thresholds identified in this cohort and to determine whether sex-stratified screening strategies improve prediabetes prevention outcomes.

## Data Availability

The data that support the findings of this study were derived from the following resource available in the public domain: China Health and Retirement Longitudinal Study (CHARLS). Access to these data is controlled due to ethical and privacy regulations. Researchers can obtain the data after registration at https://charls.pku.edu.cn, which requires signing a data use agreement. The authors confirm that they had no special access privileges to these data, and other researchers would be able to access them in the same manner as described.
