# Supplementary material for: Sex differences in the predictive value of insulin resistance surrogate indicators for prediabetes among Chinese adults aged 18–45 years: a multicenter cohort study
Source: Front Endocrinol (Lausanne). 2026 Jun 24;17:1872088. doi: 10.3389/fendo.2026.1872088 (PMC13341433; doi:10.3389/fendo.2026.1872088)
Supplement: Supplementary file 1 [file DataSheet1.docx]

**Table S1. Variance inflation factors for multicollinearity assessment in the initial model.**

| Variable | VIF Value | |
| --- | --- | --- |
|  | Step1 | Step2 |
| Age (years) | 1.1 | 1.1 |
| Gender | 3.1 | 3.1 |
| SBP (mmHg) | 2.1 | 2.1 |
| DBP (mmHg) | 2 | 2 |
| BMI (kg/m2) | 1.5 | 1.5 |
| FPG (mmol/L) | 1.0 | 1.0 |
| TC (mmol/L) | 7.7 | NA |
| TG (mmol/L) | 1.7 | 1.4 |
| HDL-C (mmol/L) | 1.7 | 1.2 |
| LDL-C(mmol/L) | 6.5 | 1.1 |
| ALT (U/L) | 1.3 | 1.3 |
| BUN (mmol/L) | 1.2 | 1.1 |
| Scr (μmol/L) | 2.4 | 2.4 |
| Smoking Status | 1.2 | 1.2 |
| Drinking Status | 1.2 | 1.2 |
| Family History of diabetes | 1.0 | 1.0 |

Abbreviations: BMI, body-mass index; FPG, fasting plasma glucose; ALT, alanine aminotransferase; SBP, systolic blood pressure; Scr, serum creatinine; DBP, diastolic blood pressure; BUN, blood urea nitrogen; TC, total cholesterol; HDL-C, high-density lipoprotein cholesterol. LDL-C, low-density lipoprotein cholesterol.

Note: Variance inflation factor (VIF) was calculated as VIF = 1/(1-R²), where R² was obtained from the regression of each variable on all other variables. Variables are arranged in descending order of VIF values. A VIF ≥ 5 was considered to indicate significant multicollinearity. In this initial assessment, all variables showed VIF values < 5, suggesting no significant multicollinearity among the variables.

**Table S2. Participant characteristics across TyG index quartiles**

| Characteristics | Q1(n=15,894) | Q2 (n=15,805) | Q3 (n=16,056) | Q4 (n=16,040) | *P*-value |
| --- | --- | --- | --- | --- | --- |
| Age, years | 34.29 ± 5.30 | 34.74 ± 5.29 | 35.24 ± 5.25 | 36.20 ± 5.08 | <0.001 |
| BMI, kg/m^2^ | 20.97 ± 2.45 | 21.77 ± 2.81 | 22.91 ± 3.06 | 24.74 ± 3.00 | <0.001 |
| SBP, mmHg | 110.15 ± 12.66 | 112.57 ± 13.12 | 115.61 ± 13.50 | 120.18 ± 13.94 | <0.001 |
| DBP, mmHg | 68.74 ± 8.99 | 70.32 ± 9.21 | 72.20 ± 9.60 | 75.68 ± 10.26 | <0.001 |
| FPG, mmol/L | 4.56 ± 0.50 | 4.71 ± 0.46 | 4.80 ± 0.44 | 4.90 ± 0.43 | <0.001 |
| TC, mmol/L | 4.25 ± 0.70 | 4.45 ± 0.74 | 4.63 ± 0.78 | 4.98 ± 0.86 | <0.001 |
| TG, mmol/L | 0.55 ± 0.12 | 0.81 ± 0.12 | 1.15 ± 0.17 | 2.12 ± 0.77 | <0.001 |
| HDL-C, mmol/L | 1.49 ± 0.29 | 1.44 ± 0.29 | 1.36 ± 0.28 | 1.25 ± 0.26 | <0.001 |
| LDL-C, mmol/L | 2.39 ± 0.52 | 2.56 ± 0.57 | 2.71 ± 0.62 | 2.88 ± 0.68 | <0.001 |
| ALT, U/L | 13.00 (10.30-17.90) | 14.80 (11.00-21.00) | 18.00 (12.80-27.00) | 26.30 (17.60-40.00) | <0.001 |
| BUN, mmol/L | 4.44 ± 1.10 | 4.41 ± 1.09 | 4.47 ± 1.09 | 4.59 ± 1.08 | <0.001 |
| Scr, μmol/L | 63.47 ± 13.67 | 67.01 ± 14.85 | 70.70 ± 15.41 | 75.85 ± 14.79 | <0.001 |
| Gender, n (%) |  |  |  |  | <0.001 |
| Male | 4245 (26.71%) | 6508 (41.18%) | 9264 (57.70%) | 12668 (78.98%) |  |
| Female | 11649 (73.29%) | 9297 (58.82%) | 6792 (42.30%) | 3372 (21.02%) |  |
| Smoking Status, n (%) |  |  |  |  | <0.001 |
| Current smoker | 831 (5.23%) | 1389 (8.79%) | 2119 (13.20%) | 3487 (21.74%) |  |
| Ever smoker | 259 (1.63%) | 407 (2.58%) | 650 (4.05%) | 788 (4.91%) |  |
| Never smoker | 14804 (93.14%) | 14009 (88.64%) | 13287 (82.75%) | 11765 (73.35%) |  |
| Drinking Status, n (%) |  |  |  |  | <0.001 |
| Current drinker | 78 (0.49%) | 127 (0.80%) | 221 (1.38%) | 429 (2.67%) |  |
| Ever drinker | 1340 (8.43%) | 1879 (11.89%) | 2592 (16.14%) | 3561 (22.20%) |  |
| Never drinker | 14476 (91.08%) | 13799 (87.31%) | 13243 (82.48%) | 12050 (75.12%) |  |
| Family History of Diabetes, n (%) |  |  |  |  | 0.016 |
| No | 15557 (97.88%) | 15454 (97.78%) | 15643 (97.43%) | 15635 (97.48%) |  |
| Yes | 337 (2.12%) | 351 (2.22%) | 413 (2.57%) | 405 (2.52%) |  |
| Prediabetes accident |  |  |  |  | <0.001 |
| No | 15152 (95.33%) | 14789 (93.57%) | 14693 (91.51%) | 13857 (86.39%) |  |
| Yes | 742 (4.67%) | 1016 (6.43%) | 1363 (8.49%) | 2183 (13.61%) |  |
| Follow-up (years) | 3.02 (2.14-4.01) | 2.97 (2.11-3.95) | 2.96 (2.12-3.89) | 2.95 (2.11-3.79) | <0.001 |

Values are n (%), means or medians (quartiles)

BMI, body-mass index; FPG, fasting plasma glucose; SBP, systolic blood pressure; DBP, diastolic blood pressure; TC, total cholesterol; TG, triglycerides; HDL-C, high-density lipoprotein cholesterol; LDL-C, low-density lipoprotein cholesterol; ALT, alanine aminotransferase; BUN, blood urea nitrogen; Scr, serum creatinine; TyG, triglyceride–glucose index.

**Table S3. Participant characteristics across TyG-BMI** **quartiles**

| Characteristics | Q1(n=15,927) | Q2 (n=15,920) | Q3 (n=15,979) | Q4 (n=15,969) | *P*-value |
| --- | --- | --- | --- | --- | --- |
| Age, years | 33.70 ± 5.18 | 34.94 ± 5.30 | 35.64 ± 5.23 | 36.20 ± 5.07 | <0.001 |
| BMI, kg/m^2^ | 19.06 ± 1.24 | 21.31 ± 1.11 | 23.39 ± 1.26 | 26.64 ± 2.17 | <0.001 |
| SBP, mmHg | 108.57 ± 12.06 | 111.73 ± 12.64 | 116.23 ± 13.00 | 122.03 ± 13.73 | <0.001 |
| DBP, mmHg | 68.17 ± 8.61 | 69.71 ± 8.99 | 72.39 ± 9.33 | 76.70 ± 10.31 | <0.001 |
| FPG, mmol/L | 4.62 ± 0.48 | 4.72 ± 0.46 | 4.78 ± 0.47 | 4.86 ± 0.46 | <0.001 |
| TC, mmol/L | 4.34 ± 0.74 | 4.46 ± 0.76 | 4.62 ± 0.80 | 4.90 ± 0.85 | <0.001 |
| TG, mmol/L | 0.68 ± 0.25 | 0.88 ± 0.33 | 1.18 ± 0.49 | 1.89 ± 0.90 | <0.001 |
| HDL-C, mmol/L | 1.52 ± 0.30 | 1.44 ± 0.28 | 1.33 ± 0.27 | 1.25 ± 0.26 | <0.001 |
| LDL-C, mmol/L | 2.43 ± 0.56 | 2.56 ± 0.58 | 2.70 ± 0.62 | 2.86 ± 0.66 | <0.001 |
| ALT, U/L | 12.50 (10.00-16.10) | 14.00 (11.00-19.60) | 18.80 (13.50-27.00) | 29.00 (19.80-43.80) | <0.001 |
| BUN, mmol/L | 4.31 ± 1.07 | 4.39 ± 1.09 | 4.54 ± 1.10 | 4.66 ± 1.08 | <0.001 |
| Scr, μmol/L | 62.13 ± 12.83 | 66.29 ± 15.04 | 72.10 ± 15.22 | 76.57 ± 14.30 | <0.001 |
| Gender, n (%) |  |  |  |  | <0.001 |
| Male | 3566 (22.39%) | 6100 (38.32%) | 9995 (62.55%) | 13024 (81.56%) |  |
| Female | 12361 (77.61%) | 9820 (61.68%) | 5984 (37.45%) | 2945 (18.44%) |  |
| Smoking Status, n (%) |  |  |  |  | <0.001 |
| Current smoker | 805 (5.05%) | 1316 (8.27%) | 2253 (14.10%) | 3452 (21.62%) |  |
| Ever smoker | 184 (1.16%) | 354 (2.22%) | 657 (4.11%) | 909 (5.69%) |  |
| Never smoker | 14938 (93.79%) | 14250 (89.51%) | 13069 (81.79%) | 11608 (72.69%) |  |
| Drinking Status, n (%) |  |  |  |  | <0.001 |
| Current drinker | 76 (0.48%) | 127 (0.80%) | 223 (1.40%) | 429 (2.69%) |  |
| Ever drinker | 1086 (6.82%) | 1729 (10.86%) | 2784 (17.42%) | 3773 (23.63%) |  |
| Never drinker | 14765 (92.70%) | 14064 (88.34%) | 12972 (81.18%) | 11767 (73.69%) |  |
| Family History of Diabetes, n (%) |  |  |  |  | 0.064 |
| No | 15591 (97.89%) | 15548 (97.66%) | 15573 (97.46%) | 15577 (97.55%) |  |
| Yes | 336 (2.11%) | 372 (2.34%) | 406 (2.54%) | 392 (2.45%) |  |
| Prediabetes accident |  |  |  |  | <0.001 |
| No | 15272 (95.89%) | 14998 (94.21%) | 14516 (90.84%) | 13705 (85.82%) |  |
| Yes | 655 (4.11%) | 922 (5.79%) | 1463 (9.16%) | 2264 (14.18%) |  |
| Follow-up (years) | 3.00 (2.14-3.98) | 2.98 (2.12-3.95) | 2.97 (2.11-3.94) | 2.95 (2.11-3.88) | <0.001 |

Values are n (%), means or medians (quartiles). BMI, body-mass index; FPG, fasting plasma glucose; SBP, systolic blood pressure; DBP, diastolic blood pressure; TC, total cholesterol; TG, triglycerides; HDL-C, high-density lipoprotein cholesterol; LDL-C, low-density lipoprotein cholesterol; ALT, alanine aminotransferase; BUN, blood urea nitrogen; Scr, serum creatinine; TyG, triglyceride–glucose index; TyG-BMI, triglyceride–glucose–body-mass index.

**Table S4. Participant characteristics across AIP quartiles**

| Characteristics | Q1(n=15,940) | Q2 (n=15,315) | Q3 (n=16,327) | Q4 (n=16,213) | *P*-value |
| --- | --- | --- | --- | --- | --- |
| Age, years | 34.41 ± 5.37 | 34.70 ± 5.33 | 35.13 ± 5.21 | 36.20 ± 5.03 | <0.001 |
| BMI, kg/m^2^ | 20.83 ± 2.38 | 21.76 ± 2.79 | 22.92 ± 3.02 | 24.82 ± 2.95 | <0.001 |
| SBP, mmHg | 110.10 ± 12.59 | 112.77 ± 13.28 | 115.56 ± 13.67 | 119.96 ± 13.76 | <0.001 |
| DBP, mmHg | 68.67 ± 8.91 | 70.36 ± 9.32 | 72.19 ± 9.64 | 75.63 ± 10.19 | <0.001 |
| FPG, mmol/L | 4.68 ± 0.46 | 4.74 ± 0.46 | 4.76 ± 0.48 | 4.80 ± 0.48 | <0.001 |
| TC, mmol/L | 4.42 ± 0.74 | 4.45 ± 0.76 | 4.57 ± 0.80 | 4.88 ± 0.87 | <0.001 |
| TG, mmol/L | 0.56 ± 0.13 | 0.82 ± 0.15 | 1.14 ± 0.23 | 2.08 ± 0.79 | <0.001 |
| HDL-C, mmol/L | 1.62 ± 0.29 | 1.45 ± 0.24 | 1.32 ± 0.22 | 1.17 ± 0.22 | <0.001 |
| LDL-C, mmol/L | 2.46 ± 0.55 | 2.55 ± 0.57 | 2.68 ± 0.62 | 2.85 ± 0.68 | <0.001 |
| ALT, U/L | 13.00 (10.28-17.30) | 14.60 (11.00-21.00) | 17.90 (12.80-27.00) | 26.60 (18.00-40.00) | <0.001 |
| BUN, mmol/L | 4.44 ± 1.09 | 4.41 ± 1.09 | 4.49 ± 1.11 | 4.57 ± 1.07 | <0.001 |
| Scr, μmol/L | 62.37 ± 13.09 | 67.02 ± 14.91 | 71.33 ± 15.62 | 76.14 ± 14.34 | <0.001 |
| Gender, n (%) |  |  |  |  | <0.001 |
| Male | 3572 (22.41%) | 6232 (40.69%) | 9727 (59.58%) | 13154 (81.13%) |  |
| Female | 12368 (77.59%) | 9083 (59.31%) | 6600 (40.42%) | 3059 (18.87%) |  |
| Smoking Status, n (%) |  |  |  |  | <0.001 |
| Current smoker | 697 (4.37%) | 1240 (8.10%) | 2202 (13.49%) | 3687 (22.74%) |  |
| Ever smoker | 212 (1.33%) | 397 (2.59%) | 652 (3.99%) | 843 (5.20%) |  |
| Never smoker | 15031 (94.30%) | 13678 (89.31%) | 13473 (82.52%) | 11683 (72.06%) |  |
| Drinking Status, n (%) |  |  |  |  | <0.001 |
| Current drinker | 85 (0.53%) | 134 (0.87%) | 254 (1.56%) | 382 (2.36%) |  |
| Ever drinker | 1259 (7.90%) | 1930 (12.60%) | 2671 (16.36%) | 3512 (21.66%) |  |
| Never drinker | 14596 (91.57%) | 13251 (86.52%) | 13402 (82.08%) | 12319 (75.98%) |  |
| Family History of Diabetes, n (%) |  |  |  |  | 0.051 |
| No | 15595 (97.84%) | 14967 (97.73%) | 15938 (97.62%) | 15789 (97.38%) |  |
| Yes | 345 (2.16%) | 348 (2.27%) | 389 (2.38%) | 424 (2.62%) |  |
| Prediabetes accident |  |  |  |  | <0.001 |
| No | 15125 (94.89%) | 14273 (93.20%) | 14903 (91.28%) | 14190 (87.52%) |  |
| Yes | 815 (5.11%) | 1042 (6.80%) | 1424 (8.72%) | 2023 (12.48%) |  |
| Follow-up (years) | 2.97 (2.11-3.97) | 2.97 (2.11-3.94) | 2.97 (2.12-3.92) | 2.99 (2.14-3.95) | <0.001 |

Values are n (%), means or medians (quartiles)

BMI, body-mass index; FPG, fasting plasma glucose; SBP, systolic blood pressure; DBP, diastolic blood pressure; TC, total cholesterol; TG, triglycerides; HDL-C, high-density lipoprotein cholesterol; LDL-C, low-density lipoprotein cholesterol; ALT, alanine aminotransferase; BUN, blood urea nitrogen; Scr, serum creatinine. AIP, atherogenic index of plasma.

**Table S5. Participant characteristics across** **METS-IR quartiles**

| Characteristics | Q1(n=15,775) | Q2 (n=15,589) | Q3 (n=16,333) | Q4 (n=16,098) | *P*-value |
| --- | --- | --- | --- | --- | --- |
| Age, years | 34.50 ± 5.33 | 34.76 ± 5.35 | 35.25 ± 5.30 | 35.95 ± 5.02 | <0.001 |
| BMI, kg/m^2^ | 20.27 ± 2.10 | 21.76 ± 2.55 | 23.18 ± 2.82 | 25.12 ± 2.92 | <0.001 |
| SBP, mmol/L | 109.63 ± 12.39 | 113.00 ± 13.34 | 116.22 ± 13.78 | 119.56 ± 13.73 | <0.001 |
| DBP, mmol/L | 68.82 ± 8.88 | 70.43 ± 9.42 | 72.40 ± 9.78 | 75.22 ± 10.18 | <0.001 |
| FPG, mmol/L | 4.60 ± 0.48 | 4.75 ± 0.45 | 4.81 ± 0.46 | 4.83 ± 0.48 | <0.001 |
| TC, mmol/L | 4.67 ± 0.76 | 4.50 ± 0.78 | 4.50 ± 0.81 | 4.66 ± 0.88 | <0.001 |
| TG, mmol/L | 0.72 ± 0.26 | 0.90 ± 0.38 | 1.17 ± 0.55 | 1.83 ± 0.92 | <0.001 |
| HDL-C, mmol/L | 1.74 ± 0.23 | 1.45 ± 0.14 | 1.29 ± 0.14 | 1.07 ± 0.15 | <0.001 |
| LDL-C, mmol/L | 2.59 ± 0.59 | 2.57 ± 0.60 | 2.63 ± 0.62 | 2.75 ± 0.68 | <0.001 |
| ALT, U/L | 13.00 (10.40-17.50) | 15.00 (11.00-21.40) | 18.00 (13.00-27.00) | 25.80 (17.20-39.40) | <0.001 |
| BUN, mmol/L | 4.34 ± 1.06 | 4.44 ± 1.10 | 4.54 ± 1.10 | 4.58 ± 1.08 | <0.001 |
| Scr, μmol/L | 61.86 ± 12.64 | 67.36 ± 15.09 | 72.06 ± 15.73 | 75.59 ± 14.36 | <0.001 |
| Gender, n (%) |  |  |  |  | <0.001 |
| Male | 3482 (22.07%) | 6503 (41.72%) | 9831 (60.19%) | 12869 (79.94%) |  |
| Female | 12293 (77.93%) | 9086 (58.28%) | 6502 (39.81%) | 3229 (20.06%) |  |
| Smoking Status, n (%) |  |  |  |  | <0.001 |
| Current smoker | 745 (4.72%) | 1350 (8.66%) | 2248 (13.76%) | 3483 (21.64%) |  |
| Ever smoker | 172 (1.09%) | 428 (2.75%) | 635 (3.89%) | 869 (5.40%) |  |
| Never smoker | 14858 (94.19%) | 13811 (88.59%) | 13450 (82.35%) | 11746 (72.97%) |  |
| Drinking Status, n (%) |  |  |  |  | <0.001 |
| Current drinker | 97 (0.61%) | 194 (1.24%) | 254 (1.56%) | 310 (1.93%) |  |
| Ever drinker | 1179 (7.47%) | 2032 (13.03%) | 2779 (17.01%) | 3382 (21.01%) |  |
| Never drinker | 14499 (91.91%) | 13363 (85.72%) | 13300 (81.43%) | 12406 (77.07%) |  |
| Family History of Diabetes, n (%) |  |  |  |  | 0.045 |
| No | 15437 (97.86%) | 15208 (97.56%) | 15963 (97.73%) | 15681 (97.41%) |  |
| Yes | 338 (2.14%) | 381 (2.44%) | 370 (2.27%) | 417 (2.59%) |  |
| Prediabetes accident |  |  |  |  | <0.001 |
| No | 15140 (95.97%) | 14473 (92.84%) | 14791 (90.56%) | 14087 (87.51%) |  |
| Yes | 635 (4.03%) | 1116 (7.16%) | 1542 (9.44%) | 2011 (12.49%) |  |
| Follow-up (years) | 2.97 (2.13-3.96) | 2.94 (2.10-3.87) | 2.96 (2.11-3.91) | 3.02 (2.17-3.99) | <0.001 |

Values are n (%), means or medians (quartiles)

BMI, body-mass index; FPG, fasting plasma glucose; SBP, systolic blood pressure; DBP, diastolic blood pressure; TC, total cholesterol; TG, triglycerides; HDL-C, high-density lipoprotein cholesterol; LDL-C, low-density lipoprotein cholesterol; ALT, alanine aminotransferase; BUN, blood urea nitrogen; Scr, serum creatinine; METS-IR, metabolic score for insulin resistance.

**Table S6.** **Factors influencing incident PREDIABETES analyzed by univariate Cox proportional hazards regression**

| Variable | Characteristics | HR (95% CI) | *P*-value |
| --- | --- | --- | --- |
| Age, years | 35.12 ± 5.28 | 1.03 (1.02, 1.03) | <0.0001 |
| BMI, kg/m^2^ | 22.60 ± 3.17 | 1.14 (1.13, 1.15) | <0.0001 |
| SBP, mmHg | 114.65 ± 13.83 | 1.03 (1.02, 1.03) | <0.0001 |
| DBP, mmHg | 71.75 ± 9.88 | 1.03 (1.03, 1.03) | <0.0001 |
| FPG, mmol/L | 4.75 ± 0.48 | 6.28 (5.85, 6.74) | <0.0001 |
| TC, mmol/L | 4.58 ± 0.82 | 1.15 (1.12, 1.19) | <0.0001 |
| TG, mmol/L | 1.16 ± 0.72 | 1.46 (1.42, 1.50) | <0.0001 |
| HDL-C, mmol/L | 1.39 ± 0.30 | 0.69 (0.63, 0.75) | <0.0001 |
| LDL-C, mmol/L | 2.64 ± 0.63 | 1.23 (1.18, 1.28) | <0.0001 |
| ALT, U/L | 16.90 (12.00-26.40) | 1.01 (1.01, 1.01) | <0.0001 |
| BUN, mmol/L | 4.48 ± 1.09 | 1.13 (1.10, 1.15) | <0.0001 |
| Scr, μmol/L | 69.28 ± 15.40 | 1.01 (1.01, 1.01) | <0.0001 |
| TyG index | 8.23 ± 0.55 | 2.33 (2.22, 2.43) | <0.0001 |
| TyG-BMI (×10) | 18.68 ± 3.41 | 1.15 (1.14, 1.16) | <0.0001 |
| AIP | -0.16 (-0.33-0.05) | 3.22 (2.94, 3.53) | <0.0001 |
| METS-IR | 2.20 ± 0.17 | 6.12 (5.29, 7.08) | <0.0001 |
| Gender, n (%) |  |  |  |
| Male | 32685 (51.23%) | 1.00 |  |
| Female | 31110 (48.77%) | 0.52 (0.49, 0.55) | <0.0001 |
| Smoking Status, n (%) |  |  |  |
| Current smoker | 7826 (12.27%) | 1.00 |  |
| Ever smoker | 2104 (3.30%) | 0.91 (0.79, 1.05) | 0.2152 |
| Never smoker | 53865 (84.43%) | 0.67 (0.62, 0.72) | <0.0001 |
| Drinking Status, n (%) |  |  |  |
| Current drinker | 855 (1.34%) | 1.00 |  |
| Ever drinker | 9372 (14.69%) | 0.82 (0.66, 1.01) | 0.0587 |
| Never drinker | 53568 (83.97%) | 0.61 (0.50, 0.75) | <0.0001 |
| Family History of Diabetes, n (%) |  |  |  |
| No | 62289 (97.64%) | 1.00 |  |
| Yes | 1506 (2.36%) | 1.10 (0.94, 1.28) | 0.2449 |

**Table S7. Discriminative performance of insulin-resistance indices for incident PREDIABETES across sex categories**

|  | **Variable** | **AUC** | **95%CI** | **Specificity** | **Sensitivity** |
| --- | --- | --- | --- | --- | --- |
| All | TyG index | 0.6298 | 0.6219-0.6376 | 0.6244 | 0.5720 |
|  | TyG-BMI (×10) | 0.6497 | 0.6421-0.6574 | 0.5758 | 0.6476 |
|  | AIP | 0.6074 | 0.5995-0.6153 | 0.5955 | 0.5601 |
|  | METS-IR | 0.6214 | 0.6139-0.6289 | 0.5326 | 0.6478 |
| Male | TyG index | 0.5976 | 0.5876-0.6076 | 0.5870 | 0.5583 |
|  | TyG-BMI (×10) | 0.6019 | 0.5920-0.6118 | 0.5939 | 0.5614 |
|  | AIP | 0.5637 | 0.5537-0.5738 | 0.5062 | 0.5901 |
|  | METS-IR | 0.5636 | 0.5538-0.5735 | 0.5457 | 0.5506 |
| Female | TyG index | 0.6063 | 0.5925-0.6201 | 0.7256 | 0.4255 |
|  | TyG-BMI (×10) | 0.6525 | 0.6390-0.6659 | 0.6677 | 0.5700 |
|  | AIP | 0.5893 | 0.5754-0.6031 | 0.5323 | 0.6064 |
|  | METS-IR | 0.6321 | 0.6190-0.6451 | 0.4978 | 0.6920 |


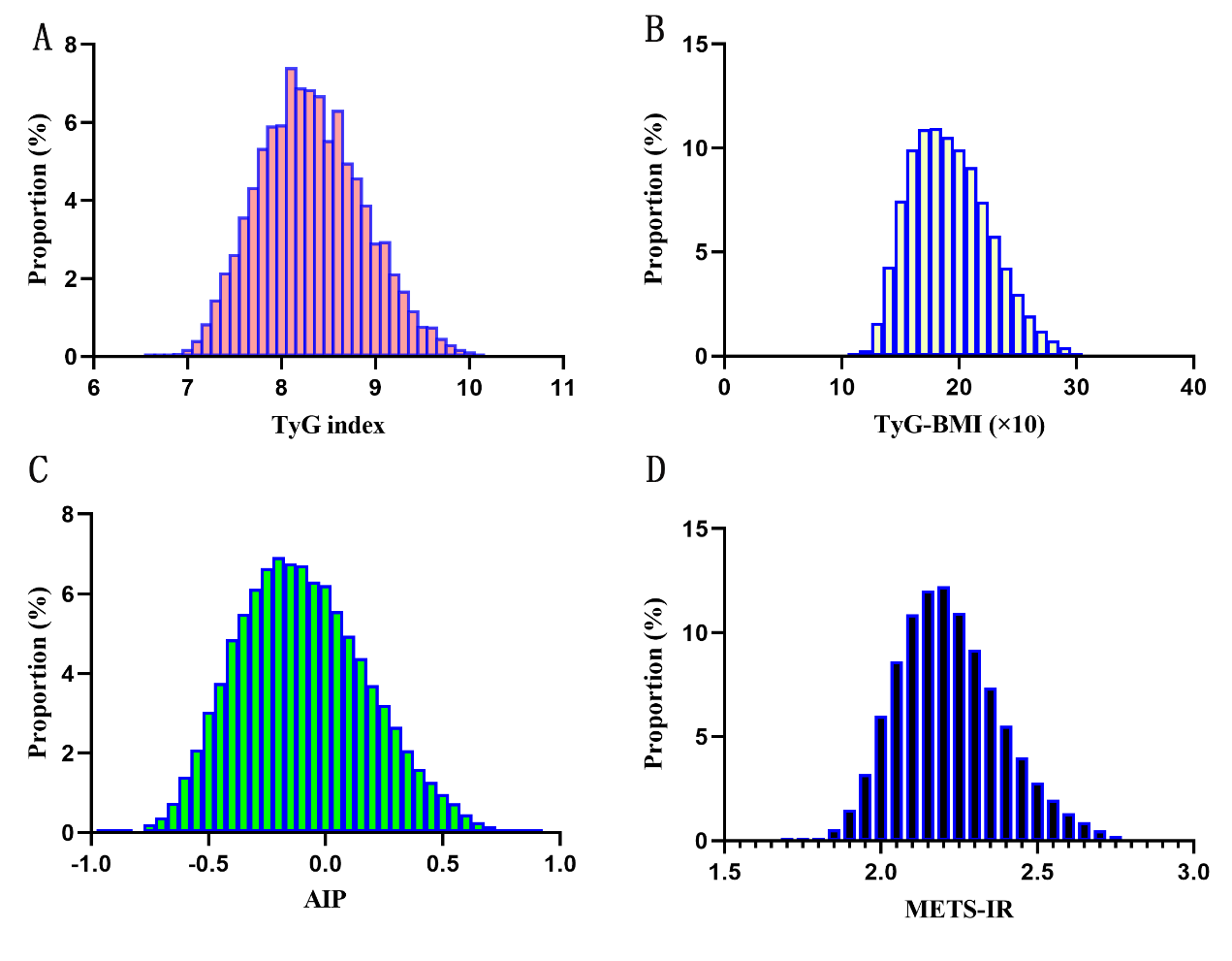


**Figure S1 Distribution of insulin resistance index.** It all presented a normal distribution, TyG index ranging from 6.6 to 10.09, with a mean of 8.32. TyG-BMI ranging from 107.7 to 299.4, with a mean of 192.27.AIP ranging from -0.95 to 0.88, with a mean of -0.1.METS-IR ranging from 1.71 to 2.75, with a mean of 2.22.


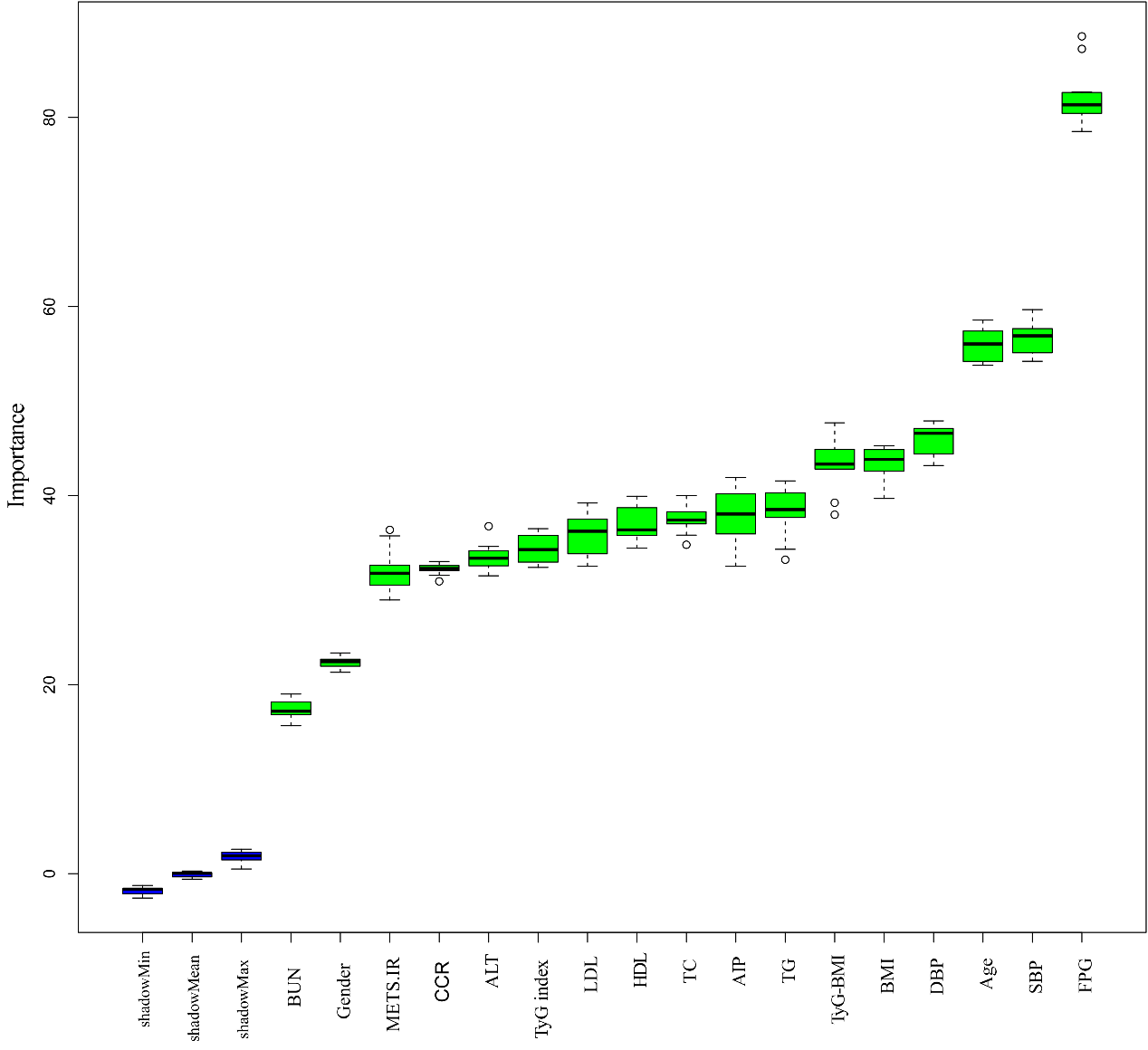


**Figure S2. The Boruta algorithm ranks the importance of potential risk factors for censor of prediabetes.** Ridge plot (Z-score) distributions graphically depict the dispersion characteristics of normalized values during model computation cycles.


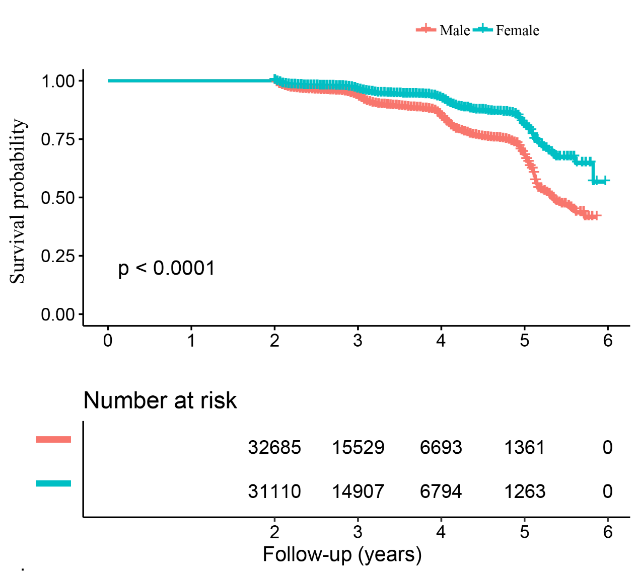


**Figure S3. Kaplan-Meier survival curves stratified by insulin resistance indices quartiles during 5-year follow-up.**


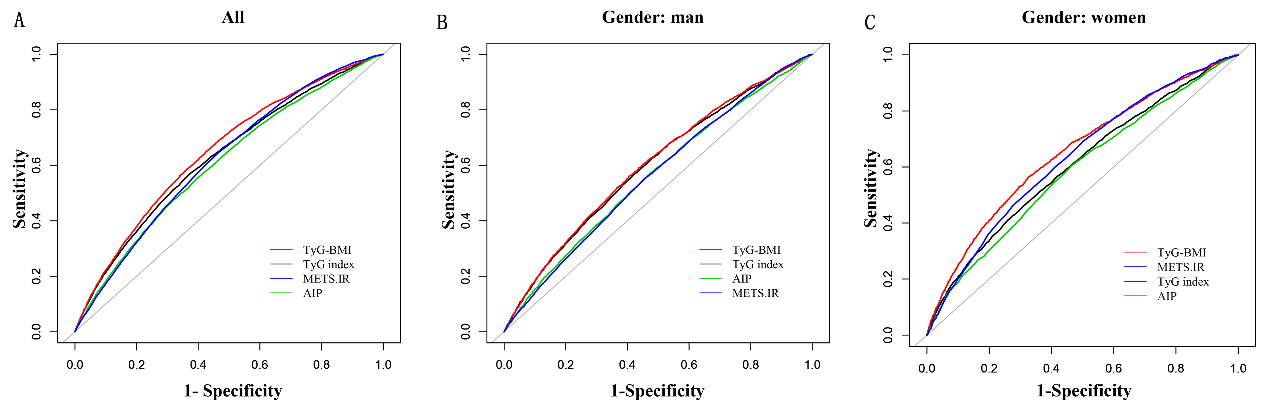


**Figure S4. ROC curves of insulin-resistance indices for predicting incident prediabetes across sex categories in the Chinese cohort.** A: all population; B: man; C: women.
